# 10061 Assessing immunogenicity of an Ebola vaccine in humans using a systems biology approach

**DOI:** 10.1017/cts.2021.626

**Published:** 2021-03-31

**Authors:** Olivia Martin, Yang Song, Sean Daugherty, Rezwan Wahidrcelo B. Sztein, Claire M. Fraser

**Affiliations:** University of Maryland

## Abstract

ABSTRACT IMPACT: Understanding gene expression changes after viral vaccination and booster may help predict vaccine efficacy. OBJECTIVES/GOALS: Utilize a systems biology approach to identify gene expression changes after administration of Zaire Ebola virus glycoprotein expressed in a Chimp Adeno3 vector (ChAd3-EBOZ) and either boosted ˜7 weeks later with modified vaccinia Ankara MVA expressing Zaire and Marburg GPs plus Tai forest NP (MVA-BN ®Filo) or given saline (placebo). METHODS/STUDY POPULATION: As part of the phase 1b, open-label vaccination trial of ChAd3-EBO-Z in Mali, West Africa, peripheral blood mononuclear cells were isolated from eight volunteers for whole genome transcriptomics analysis. Four subjects received the MVA-BN ®-Filo booster and four received saline. Samples were taken prior to receipt of the booster or placebo, as well as 1, 7, and 14 days afterwards. Significant differentially expressed genes were identified using RNA-seq between baseline and post-MVA-BN ®Filo. Functional enrichment analysis against the GO Ontology Database and the Immune Signatures C7 collection of MSigDB (ImmuneSigDB) was performed. These differentially expressed genes were also examined for associations with Ebola antibody titers and cell-mediated immune responses. RESULTS/ANTICIPATED RESULTS: The majority of gene expression changes occurred on day 1 post-MVA-BN ®-Filo administration. 870 genes had significantly different expression when day 1 samples were compared to pre-booster baseline (791 upregulated/79 downregulated). Those upregulated genes are mainly involved type I interferon and regulation of viral life cycle pathways. The downregulated genes are involved in regulation of cellular defense response, lymphocyte mediated immunity. Comparing to the C7 Immune Signatures collection datasets, we identified more than 100 upregulated genes from 6 studies of yellow fever vaccination that were also significantly upregulated in our study. The top enriched ontological pathway of those genes is cellular response to type I Interferon. DISCUSSION/SIGNIFICANCE OF FINDINGS: The use of a systems biology approach to compare gene expression changes among vaccine studies utilizing whole genome transcriptomics data allows the identification of genes involved in the immune response to vaccination and might aid in predicting vaccine efficacy.

